# The therapeutic potential of the mesenchymal stem cell secretome in ischaemic stroke

**DOI:** 10.1177/0271678X18776802

**Published:** 2018-05-17

**Authors:** Catriona J Cunningham, Elena Redondo-Castro, Stuart M Allan

**Affiliations:** Division of Neuroscience and Experimental Psychology, School of Biological Sciences, Faculty of Biology, Medicine and Health, University of Manchester, Manchester, UK

**Keywords:** Cell therapy, mesenchymal stem cell, repair, stroke, secretome

## Abstract

Mesenchymal stem cells (MSCs) hold great potential as a regenerative therapy for stroke, leading to increased repair and functional recovery in animal models of cerebral ischaemia. While it was initially hypothesised that cell replacement was an important mechanism of action of MSCs, focus has shifted to their paracrine actions or the so called “bystander” effect. MSCs secrete a wide array of growth factors, chemokines, cytokines and extracellular vesicles, commonly referred to as the MSC secretome. There is evidence suggesting the MSC secretome can promote repair through a number of mechanisms including preventing cell apoptosis, modulating the inflammatory response and promoting endogenous repair mechanisms such as angiogenesis and neurogenesis. In this review, we will discuss the in vitro approaches currently being employed to drive the MSC secretome towards a more anti-inflammatory and regenerative phenotype. We will then examine the role of the secretome in promoting repair and improving recovery in preclinical models of cerebral ischaemia.

## Introduction

Stroke is a major global health problem with limited treatment options which leads to around 6.7 million deaths annually.^1^ For the 33 million people living with stroke, a significant proportion have some disability.^2^ Current treatments for acute ischaemic stroke are based on reperfusion through thrombolysis or endovascular therapy. Both approaches are very effective and have led to significant re-organisation of acute stroke services to allow greater access to these treatments. However, due to the narrow therapeutic window for administration of tPA (< 4.5 h of symptom onset), only 5% of patients in the UK receive thrombolysis^3^ and an estimated 10% would be eligible for endovascular clot retrieval assuming national coverage,^4^ which is still not the case. Therefore, there is much interest in developing regenerative therapies to alleviate the disability caused by stroke.

One promising candidate being widely investigated as a cell therapy for ischaemic stroke is mesenchymal stem/stromal cells (MSCs), multipotent cells first described by Friedenstein and colleagues in the 1960s and 1970s.^5^ While initially found in bone marrow, MSCs have since been isolated from most postnatal organs^6^ including adipose tissue,^7^ dental pulp,^8^ lungs, liver, spleen and brain.^9,10^ MSCs are also present in foetal tissues such as placenta, umbilical cord^11^ and Wharton’s jelly.^12^ The International Society for Cellular Therapy (ISCT) has defined the minimum criteria for MSCs as: adherence to tissue culture plastic; multipotency as demonstrated by in vitro differentiation into osteoclasts, adipocytes and chondroblasts; expression of surface markers CD73, CD90 and CD105; and negative for CD34, CD45, CD14 or CD11b, C79α or CD19 and HLA-DR.^13^

A large number of clinical trials (794 as of January 2018) have been conducted or are ongoing to investigate MSCs as a potential therapy for a wide range of diseases including graft versus host disease, haematological malignancies, diabetes, and neurological diseases such as Alzheimer’s disease and amyotrophic lateral sclerosis.^14,15^ More specifically, a number of phase I/II clinical trials have suggested MSCs are a safe and feasible therapy for stroke.^16–21^ MSCs are immune evasive^22^ and less immunogenic than many other cell types due to low expression of majority histocompatibility complex class I molecules.^23^ In support of this, a meta-analysis conducted by Lalu et al.^14^ found no association between acute infusional toxicity and MSC treatment overall and no adverse events in the 13 studies that used allogeneic cells. Thus, allogeneic transplantation without immunosuppressive therapy appears to be safe which has numerous advantages over autologous therapies including decreased cost and time to administration.^23^

Numerous preclinical studies have demonstrated that treatment with stem cells, including MSCs, promotes functional recovery in rodent models of cerebral ischaemia. Although it was thought initially that the principle mechanism of therapeutic action of stem cells was direct replacement of dead and injured cells, this has been largely disregarded as very few cells reach the site of injury, engraft and survive long term.^24,25^ Following administration by intravenous (IV) or intra-arterial (IA) injection, the vast majority of MSCs become entrapped in the lungs within 48 h.^26,27^ Li et al.^28^ reported that around 4% of cells were present in the ischaemic brain of rats 14 days after tail vein injection. Additionally, only a small percentage (<10%) of transplanted MSCs differentiate and express neuronal markers such as NeuN and MAP-2.^29–32^ To further disregard the cell replacement hypothesis, MSCs lack expression of the voltage-gated ion channels required for generating action potentials.^33^ Despite this, MSC treatment leads to significant improvements in functional outcomes and can occur independently of cell migration to the ischaemic brain.^28,34^ There is growing evidence to support the paracrine actions of MSCs, also known as the bystander effect, in improving outcome in preclinical models of stroke. MSCs secrete a wide range of chemokines, cytokines, growth factors and extracellular vesicles (EVs) collectively termed the secretome.

In this review, we will firstly discuss in vitro approaches to modifying the MSC secretome to enhance a more anti-inflammatory and regenerative phenotype. We will then look at the involvement of the MSC secretome in promoting repair mechanisms, modulating inflammation and improving functional outcomes in preclinical models of cerebral ischaemia.

## Approaches to enhancing the MSC secretome

**Figure 1.**
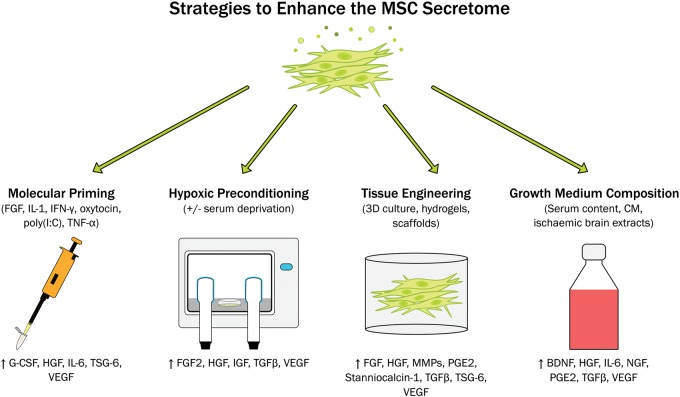
Summary of in vitro approaches that have been utilised to enhance the therapeutic potential of mesenchymal stem cell secretome. BDNF: brain-derived neurotrophic factor; FGF: fibroblast growth factor; G-CSF: granulocyte-colony stimulating factor; HGF: hepatocyte growth factor; IFN-γ: interferon gamma; IGF: insulin-like growth factor; IL: interleukin; MMPs: matrix metalloproteinases; NGF: nerve growth factor; PGE2: prostaglandin E2; TGF-β: transforming growth factor beta; TNF-α: tissue necrosis factor alpha; TSG-6: TNF-α–stimulated gene 6 protein; VEGF: vascular endothelial growth factor.

MSCs secrete numerous growth factors, chemokines and cytokines including vascular endothelial growth factor (VEGF), insulin-like growth factor 1 (IGF-1), basic fibroblast growth factor (bFGF), transforming growth factor beta-1 (TGF-β1), nerve growth factor (NGF), placental growth factor (PGF), stromal-derived growth factor (SDF-1/CXCL12), monocyte chemoattractant protein-1 (MCP-1/CCL2), interleukin-6 (IL-6), IL-8, IL-10 and IL-13.^35–38^ There is some heterogeneity in the secretome of different populations of MSCs. Adipose-derived MSCs were reported to have higher mRNA expression of VEGF-D, IGF-1 and IL-8, while dermal sheath and dermal papilla-derived cells secreted higher concentrations of CCL2 and leptin than other populations.^39^ Additionally, Du et al.^40^ found increased expression levels of HGF (hepatocyte growth factor), bFGF, IL-6, IL-8, IL-1α and IL-1β in placenta-derived MSCs and in bone marrow-derived populations, VEGF-A, NGF and angiogenin were higher. Thus, MSCs secrete a number of factors that could promote angiogenesis and neurogenesis, prevent apoptosis and modulate inflammatory responses. The MSC secretome therefore has great potential as a regenerative therapy for stroke and a number of strategies have been employed to further enhance this reparative capacity (Figure 1).

## Molecular priming

Priming or preconditioning acts as a sub-lethal event that can trigger an adaptive response to a future injury or damage. Therefore, administration of “trained” cells better able to respond to the ischaemic and inflammatory environment post-stroke may further enhance the efficacy of MSC therapies. MSCs from different sources (mainly bone marrow, adipose, placenta and umbilical cord) and from different species (human, equine, murine) have been preconditioned or primed. Such in vitro preconditioning strategies can be selective and aimed at improving the secretion of certain factors such as anti-inflammatory TNF-α-stimulated gene 6 protein (TSG-6),^41^ or to increase survival of MSC once transplanted.^42^ Non-selective approaches aim to modulate the MSC secretome towards a more desirable phenotype by inducing the secretion of immunomodulatory,^43^ anti-inflammatory^44^ or pro-angiogenic molecules.^45^

MSCs are known to be great immune modulators, so they are often used to decrease inflammatory responses. To enhance this characteristic, cells can be primed with inflammatory mediators such as IL-1,^43^ TNF-α,^41,46,47^ IFN-γ^48,49^ or combinations of these.^50^ In response to these priming stimuli, MSCs secrete higher concentrations of immunomodulatory mediators including prostaglandin E2 (PGE2), IL-6 and granulocyte-colony stimulating factor (G-CSF)^43,51^ and upregulate adhesion molecule expression.^46,50^ This leads to increased promotion of endogenous repair mechanisms including angiogenesis^46,50^ and osteogenesis^46^ which has been shown to be beneficial in in vivo models of arthritis,^48^ joint and cartilage injuries^47,50^ and bone regeneration.^46^ It has been reported though, that inflammatory priming can lead to an increased immunogenicity.^50^ As this can be detrimental in future cell therapies, short priming durations with low doses of pro-inflammatory mediators should be used to limit this undesirable effect. For example, our lab demonstrated 5 min of priming with IL-1α drove the MSC secretome towards a more anti-inflammatory phenotype which decreased secretion of TNF-α and IL-6 from inflamed mouse microglia.^43^ A wide variety of molecules can be used to prime MSCs and modify their secretome. Indeed, the screening of libraries has already become a suitable strategy to detect active molecules.^52^ As an example, polyinosinic and polycytidylic acid (poly(I:C)) can be used as a toll-like receptor 3 (TLR3) stimulus to induce an increased anti-inflammatory phenotype,^44^ while oxytocin^53^ or FGF2^45^ have been used to increase the angiogenic potential of the MSC secretome.

## Hypoxia

Another alternative to induce an improved response to ischemic environments is the use of hypoxic or ischaemic preconditioning. This has been shown to induce increased MSC proliferation and migration,^54^ upregulation of glucose transporters and adhesion molecule expression,^55^ and drive the secretome towards a pro-angiogenic phenotype.^56^ More specifically, hypoxic preconditioning of bone marrow-derived MSCs induces increased secretion of FGF2, VEGF, HGF, TGF-β and IGF.^57,58^ This has also been reported in MSCs derived from other sources including placenta^59,60^ and adipose tissue.^61^ Hypoxic preconditioning can enhance the therapeutic potential of MSCs in vivo preventing apoptosis of cardiomyocytes and promoting angiogenesis after myocardial infarction^62^ as well increasing secretion of VEGF, HGF and FGF in a murine model of critical limb ischaemia.^54^ Serum deprivation is often used in conjunction with hypoxic preconditioning as it improves the ability of MSCs to induce angiogenesis and endothelial proliferation.^63,64^

## 3D culture and biomaterials

The 3D culture of MSCs is another option to achieve a more effective therapy for ischaemic stroke.^65–67^ Culturing in 3D enhances the angiogenic potential of MSCs by increasing the secretion of molecules including VEGF, HGF and FGF2,^65,68,69^ and increases anti-inflammatory potential by secreting TSG-6, stanniocalcin-1, PGE2 or TGF-β amongst others.^65,70,71^ This 3D environment provides more physiological conditions, maintains stemness and increases cell survival and multipotency once transplanted.^65,72^ Additionally, this increases the ability of MSCs to activate endogenous mechanisms of tissue repair through increased secretion of factors such as matrix metalloproteinases (MMPs) and FGF2.^67,73^ MSC spheroids have already shown moderate success in promoting bone regeneration^74,75^ and in inflammatory models such as colitis.^76^

In some studies, biomaterials including hydrogels, assembling peptides or scaffolds have been utilised to further enhance the anti-inflammatory and pro-trophic phenotype of the MSC secretome. Murphy et al.^77^ showed that entrapping MSC spheroids in a fibril gel can increase secretion of VEGF and PGE2, increase endothelial cell proliferation and promote angiogenesis in a human 3D skin equivalent wound model. Similarly, conditioned medium (CM) derived from MSCs embedded in collagen and polyethylene glycol hydrogels induced stronger antioxidant and neuroprotective responses in SH-SY5Y cells.^78^ MSCs cultured with self-assembly peptides induced in vitro outgrowth of axons and neurites from neurons following traumatic brain injury.^79^ Combined administration of MSCs and biomaterials has been previously shown to promote repair in a number of disease models. For example, embedding MSCs in platelet lysate hydrogels increased engraftment as well as increasing the pro-angiogenic and neo-vascularisation activity of the transplanted cells in a murine model of critical limb ischaemia.^80^

## CM and serum preconditioning

When the molecule intended to trigger a particular effect is not known, or when a specific environment needs to be mimicked, CM or serum is another suitable option for modifying the MSC secretome. MSCs treated with endothelial growth medium show improved viability and endothelial-related functions,^81^ while priming MSCs with serum from stroke animals increased proliferation and secretion of cytokines, thus improving their therapeutic potential.^82^ Similarly, when cultured in rat ischaemic brain extracts, MSCs respond by increasing secretion of BDNF, VEGF, NGF and HGF.^83^ The serum content of growth medium can have a profound effect on the MSC secretome. Zimmerman and McDevit^71^ showed the secretion of immunomodulatory factors such as PGE2, IL-6 and TGF-β was far increased when MSC spheroids were cultured in growth media containing foetal bovine serum as compared with a specialised MSC serum-free medium.

## Role of the MSC secretome in promoting repair in preclinical models of stroke

There is a substantial body of evidence demonstrating MSC transplantation promotes recovery in rodent models of stroke although the mechanisms of action have not been fully elucidated. A number of studies from the early 2000s began to hypothesise that the MSC secretome was involved. Zhao et al.^84^ suggested that as intracranial (IC) administration of hMSCs one week after middle cerebral artery occlusion (MCAO) in spontaneously hypertensive (SHR) rats was associated with improvements in limb placement but differentiation was limited, recovery might be mediated through secretion of neurotrophic factors from the transplanted cells. Similarly, IV administration of MSCs also improved neurological deficits and the authors proposed neurotrophins from the MSCs decreased apoptosis and promoted endogenous neurogenesis.^28^ Later work from the same lab also showed that MSC transplantation increased angiogenesis in the ischaemic boundary.^85^ This was associated with increased endogenous VEGF and VEGF receptor 2 (VEGFR2) expression, which the authors hypothesised were upregulated by secretion of growth factors such as bFGF from the MSCs. In support of this, exogenous IGF-1 from transplanted cells has been detected in the core and ischaemic border zone three days post-MCAO, while expression of endogenous growth factors including VEGF, EGF and bFGF was increased in MSC-treated rats compared with controls.^86^ Additionally, secretion of a number of other factors from MSCs engrafted in the ischaemic brain has been detected including BDNF, bFGF, CXCL12, platelet-derived growth factor-AA (PDGF-AA) and angiopoietin-2 (Ang-2).^87,88^

One neurotrophin of particular interest is BDNF which promotes neuronal survival and differentiation through interaction with tyrosine kinase receptors.^89^ In preclinical models of stroke, IV BDNF administration reduced infarct volume, improved recovery and promoted neurogenesis.^90,91^ Furthermore, BDNF appears to be an important mediator in the MSC secretome preventing glutamate-induced neuronal death in vitro*.*^92^ When transplanted into a stroke model, BDNF secretion from MSCs was associated with increased functional recovery, decreased lesion volume, decreased apoptosis and increased angiogenesis.^34^ Several studies have shown that overexpression of BDNF in MSCs further enhanced repair and recovery.^93–95^ However, Koh et al.^96^ demonstrated that neutralising BDNF did not completely ameliorate the observed improvements in neurological function following human umbilical cord-derived MSC transplantation, suggesting other mediators are important in promoting recovery after stroke.

VEGF has both beneficial and detrimental effects in the post-stroke brain, as reviewed by Greenberg and Jin.^97^ In brief, VEGF increases neuroprotection, angiogenesis and neurogenesis after focal cerebral ischaemia^98^ but as a potent inducer of vascular permeability, can also increase blood–brain barrier (BBB) leakage leading to cerebral oedema.^99,100^ These dual actions appear to be reflected in the literature on the involvement of the MSC secretome in stroke repair. A number of studies have shown overexpression of VEGF in MSCs (VEGF-MSCs) enhanced functional recovery, decreased lesion volume, promoted neurogenesis and decreased neuronal apoptosis in rodent models of cerebral ischaemia.^101–103^ In contrast, VEGF-MSCs have also been shown to worsen functional outcomes and increase oedema, while Ang-VEGF-MSCs led to improved recovery, decreased lesion volumes and increased angiogenesis.^104^ In a cardiac arrest-induced model of global cerebral ischaemia, overexpression of both VEGF and BDNF led to decreased apoptosis and increased motor recovery.^103^ Overexpression of a plethora of other cytokines and growth factors including Ang-1, GDNF, HGF, FGF1 and PIGF were also shown to enhance recovery after cerebral ischaemia.^104–109^ Interestingly, MSCs transfected with either neurotrophin 3 (NT3) or ciliary neurotrophic factor (CNTF) did not significantly improve functional outcomes.^110^ Thus, it appears that a combination of mediators are involved in promoting functional recovery in preclinical models of ischaemic stroke as summarised in Table 1.

**Table 1.** Summary of studies investigating the efficacy of MSC therapies in preclinical models of cerebral ischaemia and the proposed involvement of secretome components.

Publication	MSC therapy	Stroke model	Route	Dose	Timing post-stroke	Results	Potential role of the secretome
Chen et al.^85^	Human BMSCs	120 min MCAO, male Wistar rats	IV	1 × 10^6^	24 h	Increased angiogenesis	bFGF
Cheng et al.^121^	Human UMSCs	90 min MCAO, male mice	IV	4 × 10^6^	30 min	Improved functional recovery (mNSS), decreased neuroinflammation, decreased infarct volume, decreased oedema	TGF-β
Deng et al.^102^	Rat BMSCs	Permanent MCAO, male Sprague-Dawley rats	IV	2 × 10^6^	2 or 24 h	Improved functional recovery (mNSS, MWM), decreased apoptosis, increased endogenous neurogenesis	VEGF
Ghazavi et al.^108^	Rat FGF-ADMSCs	30 min MCAO, male Wistar rats	IV	2 × 10^6^	30 min	Improved functional recovery (rotarod, Roger’s test), decreased infarct volume, decreased apoptosis	FGF1
Guo et al.^72^	Human PMSCs, 3D cultured, dissociated	120 min MCAO, female Sprague-Dawley rats	IA	1 × 10^6^	24 h	Increased functional recovery (mNSS, adhesive removal), decreased lesion volume, increased angiogenesis	VEGF, bFGF
Horita et al.^105^	Human GDNF-BMSCs	Permanent MCAO, male Sprague-Dawley rats	IV	1 × 10^7^	3 h	Improved functional recovery (treadmill stress test), decreased infarct volume	GDNF
Ishizaka et al.^34^	Human MSCs	75 min MCAO, male Sprague-Dawley rats	IA	1 × 10^6^	1, 4 or 7 days	Improved functional recovery (cylinder test), decreased brain atrophy, increased angiogenesis, decreased activated microglial recruitment	BDNF
Jeong et al.^95^	Human BDNF-BMSCs	90 min MCAO, male Sprague-Dawley rats	IC	5 × 10^5^	3 days	Improved functional recovery (adhesive removal, rotarod), decreased infarct volume, decreased apoptosis, increased endogenous neurogenesis	BDNF
Koh et al.^96^	Human UCMSCs	120 min MCAO, male Sprague-Dawley rats	IC	6 × 10^5^	2 weeks	Improved functional recovery (NDS), decreased infarct volume, increased endogenous neurogenesis	BDNF
Kurozumi et al.^93^	Human BDNF-BMSCs	90 min MCAO, male Wistar rats	IC	5 × 10^5^	24 h	Improved functional recovery (limb placement, treadmill test), decreased infarct volume, decreased apoptosis	BDNF
Kurozumi et al.^110^	Human BDNF/GDNF/ CNTF/NT3-BMSCs	90 min MCAO, male Wistar rats	IC	5 × 10^5^	24 h	Improved functional recovery (limb placement, treadmill test), decreased infarct volume	BDNF, GDNF
Li et al.^28^	Human BMSCs	120 min MCAO, male Wistar rats	IV	1 × 10^6^	24 h	Improved functional recovery (adhesive removal, mNSS)	Neurotrophins
Lin et al.^87^	Human UMSCs	90 min MCAO, male Sprague-Dawley rats	IC	5 × 10^5^	24 h	Improved functional recovery (rotarod), decreased cortical atrophy	BDNF, bFGF, PDGF-AA, Ang-2, CXCL16, neutrophil-activating protein-2
Lin et al.^130^	Rat BMSCs	CA global cerebral ischaemia, male Sprague-Dawley rats	IV	5 × 10^6^	2 h	Improved functional recovery (adhesive removal, rotarod), decreased neuroinflammation	TSG-6
Liu et al.^109^	Human PGF-BMSCs	Permanent MCAO, male Sprague-Dawley rats	IV	1 × 10^7^	3 h	Improved functional recovery (treadmill stress test, limb placement test), decreased infarct volume, decreased apoptosis, increased angiogenesis	PGF
Miki et al.^101^	Rat VEGF-BMSCs	120 min MCAO, male Wistar rats	IC	1 × 10^6^	24 h	Improved functional recovery (mNSS), decreased infarct volume, decreased neuronal apoptosis	VEGF
Nakajima et al.^125^	Human IL-10-BMSCs	90 min MCAO, male Sprague-Dawley rats	IV	1 × 10^6^	0 or 3 h	Improved functional recovery (NDS, rotarod), decreased infarct volume, decreased neuroinflammation, decreased neuronal degeneration	IL-10
Nomura et al.^94^	Human BDNF-BMSCs	Permanent MCAO, male Sprague-Dawley rats	IV	1 × 10^7^	6 h	Improved functional recovery (treadmill stress test), decreased infarct volume	BDNF
Onda et al.^107^	Human Ang-VEGF- BMSCs	Permanent MCAO, male Sprague-Dawley rats	IV	1 × 10^6^	6 h	Improved functional recovery (treadmill stress test), decreased infarct volume, increased angiogenesis	Ang-1
Shichinohe et al.^92^	Mouse BMSCs	Permanent MCAO, male Balb/c mice	IC	2 × 10^5^	1 week	Improved survival of neurons in per-infarct	BDNF
Sheikh et al.^128^	Human BMSCs	90 min MCAO, male Wistar rats	IV	3 × 10^6^	24 h	Decreased microglial activation and proinflammatory gene expression	IL-5, CX3CL1
Toyama et al.^104^	Human Ang/VEGF/ Ang-VEGF-BMSCs	Permanent MCAO, male Sprague-Dawley rats	IV	1 × 10^6^	6 h	Improved functional recovery (treadmill stress test), decreased infarct volume, increased angiogenesis	Ang-1, VEGF
Toyoshima et al.^88^	Rat BMSCs	90 min MCAO, male Wistar rats	IA	1 × 10^6^	1, 6, 24 or 48 h	Improved functional recovery (mNSS), decreased infarct volume	bFGF, CXCL12
Wakabayashi et al.^86^	Human BMSCs	60 min MCAO, male Wistar rats	IV	3 × 10^6^	24 h	Improved functional recovery (mNSS), decreased infarct volume	IGF-1
Wei et al.^131^	Rat BMSCs, hypoxic preconditioning	90 min MCAO, male Wistar rats	IV	1 × 10^6^	24 h	Increased functional recovery (rotarod), increased angiogenesis, decreased microglial activation	HIF-1α, BDNF, GDNF, VEGF, CXCL12
Yoo et al.^122^	Human BMSCs	120 min MCAO, male Sprague-Dawley rats	IC	5 × 10^5^	3 days	Improved functional recovery (adhesive removal, rotarod), decreased neuroinflammation, decreased infarct volume	TGF-β
Zacharek et al.^132^	Rat BMSCs isolated post-MCAO	120 min MCAO, male Wistar rats	IV	1 × 10^6^	24 h	Increased functional recovery (mNSS, foot fault), increased angiogenesis	Ang1, bFGF, GDNF, VEGF
Zhao et al.^84^	Human BMSCs	Permanent MCAO, male SHR rats	IC	1 × 10^6^	1 week	Improved functional recovery (limb placement)	Neurotrophins
Zhao et al.^106^	Rat HGF-BMSCs	120 min MCAO, male Wistar rats	IV	1 × 10^6^	24 h	Improved functional recovery (mNSS), decreased infarct volume, decreased neuronal apoptosis	HGF
Zhou et al.^103^	Rat BDNF-VEGF- BMSCs	CA global cerebral ischaemia, male Sprague-Dawley rats	IV	3 × 10^6^	2 h	Improved functional recovery (NDS), decreased apoptosis, increased angiogenesis	BDNF, VEGF

BMSCs: bone marrow-derived mesenchymal stem cells; CA: cardiac arrest; IN: intranasal: MCAO: middle cerebral artery occlusion; MWM: Morris water maze; mNSS: modified neurological severity score; NDS: neurological deficit score; PMSCs: placenta-derived mesenchymal stem cells; UMSCs: umbilical cord-derived mesenchymal stem cells.

## Immunomodulation

While the consensus in the literature is that the MSC secretome promotes recovery after stroke through mechanisms including neuroprotection, neurogenesis and angiogenesis after stroke, its role in immunomodulation is not clear. MSCs exert numerous immunomodulatory effects on immune cell populations including inhibition of proliferation of natural killer (NK) cells,^111^ inhibition of both B and T cell proliferation^112–114^ and suppression of dendritic cell (DC) differentiation and migration.^115,116^ Additionally, co-culture of MSCs drives the secretome of DCs, T cells, macrophages and NK cells towards anti-inflammatory phenotypes.^117,118^ A number of molecules secreted by MSCs including PGE2, TSG-6, TGF-β, HGF and IL-10 have been implicated in mediating these immunosuppressive effects.^119,120^ For example, Di Nicola et al.^113^ showed TGF-β and HGF secretion was involved in MSC suppression of T-lymphocyte proliferation.^113^ Following on from this, TGF-β secretion from transplanted MSCs improved the systemic inflammatory response after stroke decreasing Th17 cells and increasing regulatory T cells in the peripheral immune system.^121^ This was associated with decreased infarct volume and improved functional recovery. Furthermore, transplantation of TGF-β silenced MSCs did not decrease CD68+ cell infiltration or prevent microglial cell death as demonstrated in non-modified cells.^122^

IL-10, often referred to as an anti-inflammatory cytokine, is an inducer of immune tolerance and has previously been shown to have neuroprotective effects and decrease pro-inflammatory signalling in preclinical models of cerebral ischaemia.^123,124^ Transplantation of MSCs overexpressing IL-10 led to decreased microglial activation and pro-inflammatory cytokine (IL-6, TNF-α and IL-1β) concentrations in the brain after stroke compared with non-modified MSCs and vehicle.^125^ Administration of IL-10-MSCs was also neuroprotective leading to decreased neuronal degeneration and improved functional recovery. CX3CL1 (fractalkine) may also have a role in immunomodulation after cerebral ischaemia. Its receptor CX3CR1 is expressed by microglia and CX3CL1-CX3CR1 signalling supresses neurotoxic microglia activity.^126^ Secretion of CXC3CL1 from MSCs has previously been shown to shift microglia towards a neuroprotective phenotype.^127^ Sheikh et al.^128^ suggested CX3CL1 and IL-5 were involved in decreasing microglial activation and inhibiting expression of pro-inflammatory gene expression, namely COX-2 and iNOS, in the core and ischaemic border zone.

TSG-6 secretion from MSCs has previously been shown to decrease inflammation in peritonitis and corneal injury models.^41,129^ MSC administration in a cardiac arrest-induced global cerebral ischaemia rat model led to decreased serum pro-inflammatory cytokines and S100B concentrations and decreased expression of neutrophil elastase in the cerebral cortex.^130^ While TSG-6 expression in the cerebral cortex was upregulated, it was not possible to determine whether this was due to secretion from the MSCs or endogenous cells.

## Secretome modification

As discussed earlier, a number of in vitro strategies have been utilised to enhance the MSC secretome but few have investigated whether these lead to enhanced recovery of function in preclinical models of cerebral ischaemia. Transplantation of hypoxic preconditioned MSCs was superior to normoxic-treated cells leading to larger improvements in functional recovery, increased angiogenesis and decreased microglial activation.^131^ The authors proposed this was mediated by enhanced secretion of trophic factors and reported upregulated expression of BDNF, VEGF, GDNF, and CXCL12 in hypoxic cells. Similarly, Zacharek et al.^132^ demonstrated that MSCs isolated from rats after MCAO provided a better allogeneic stroke therapy compared with cells from naïve animals and was associated with increased Ang1, bFGF, GDNF and VEGF expression. The 3D culture of MSCs has also been shown to enhance recovery. MSCs cultured as spheroids and then dissociated prior to IA administration led to improved functional outcomes, increased angiogenesis and decreased lesion volume.^72^

## MSC CM treatment

In further support of the important role of the MSC secretome, CM has also been shown to promote recovery in rodent models of cerebral ischaemia. Egashira et al.^133^ reported that adipose-derived hMSC CM administered by intracerebroventricular (ICV) injection 1 h prior to MCAO in inbred DDY mice led to decreased lesion volume and neurological deficits at 24 h post-stroke. Additionally, delayed administration of CM from spheroid cultured cells beginning at day 8 post-stroke led to decreased microglial apoptosis, increased endothelial cell proliferation and improved rotarod performance at day 15.^134^ IV^135^ and intranasal^136^ administration of CM has also been reported to improve recovery.

## MSC-derived EVs

In very recent years, preclinical studies have begun demonstrating the role of mesenchymal stem cell-derived EVs in stroke repair (summarised in Table 2). MSCs secrete a number of EVs including exosomes which are characteristically 30–100 nm in diameter and contain micro RNAs, messenger RNAs and proteins.^137^ Microvesicles (MVs), also known as shedding vesicles, ectosomes or microparticles, ranging from 60 nm to 1 µm in diameter are also secreted.^138^ Systemic administration of EVs derived from MSCs has been shown to promote functional recovery in rodent models of cerebral ischaemia and this was associated with mechanisms including neuroprotection, white matter repair, neurogenesis and angiogenesis.^139–142^ In a transient global ischaemia model, exosome therapy also ameliorated impairments in memory and hippocampal synaptic transmission.^143^ Furthermore, MSC-derived exosomes have been shown to be equally effective as MSCs in improving functional outcomes, further supporting the importance of the secretome in promoting stroke repair.^140^ Lee et al.^141^ showed that MVs derived from MSCs preconditioned with either normal or ischaemic brain extracts further enhanced recovery compared with MVs from untreated cells. There are a limited number of studies postulating on the role of specific EVs in stroke repair. Overexpression of miR-133b^144,145^ and miR-17-92 cluster^146^ was associated with increased functional recovery and repair. In a diabetic mouse model, miR-126 was shown to promote functional recovery, angiogenesis, white matter remodelling and decrease BBB permeability.^147^

**Table 2.** Preclinical studies on the effect of MSC-derived exosomes on repair and recovery after ischaemic stroke.

Publication	Exosome therapy	Stroke model	Route	Dose	Timing post-stroke	Results
Chen et al.^147^	Human UMSCs/UMSCs MiR-126^−/−^	Distal MCAO, male db/db mice	IV	1 × 10^6^ cells	3 days	Increased functional recovery (adhesive removal, food pellet catching), decreased haemorrhagic transformation, decreased BBB permeability, increased vascular and white matter remodelling
Deng et al.^143^	Mouse BMSC-derived EVs	Transient global cerebral ischaemia, male C57Bl/6 mice	IC	200 µg	0 h	Improved cognitive impairment (MWM), improved synaptic transmission and long-term potentiation
Doeppner et al.^140^	Human BMSC-derived exosomes	30 min MCAO, male C57Bl/6 mice	IV	From 2 × 10^6^ cells	1, 3 and 5 days	Increased functional recovery (rotarod, tightrope, corner turn), neuroprotection, angiogenesis, neurogenesis, decreased immunosuppression
Otero-Ortega et al.^142^	Rat AMSC-derived exosomes	Endothelin-1 SCI, male Sprague-Dawley rats	IV	100 µg	24 h	Increased functional recovery (beam walking, Rogers test, rotarod), reduced lesion volume, increased axonal sprouting, oligodendrogenesis, remyelination and fibre tract integrity
Lee et al.^141^	Human MSC-derived MVs, treated with normal/ischaemic brain extract	Permanent MCAO, male Sprague-Dawley rats	IA	0.2 mg/kg	48 h	Increased functional recovery (torso twisting test, open field, balance beam, prehensile traction score, mNSS), decreased inflammation, decreased lesion volume, increased neurogenesis, angiogenesis
Xin et al.^139^	Rat BMSC-derived exosomes	120 min MCAO, male Wistar rats	IV	100 µg	24 h	Increased functional recovery (mNSS, foot fault), neurite remodelling, neurogenesis and angiogenesis
Xin et al.^144^	Rat BMSCs wt/MiR-133b^−^/ BMSCs MiR-133b^+^	120 min MCAO, male Wistar rats	IV	3 × 10^6^ cells	24 h	Increased functional recovery (adhesive removal, foot fault), increased axonal plasticity and neurite remodelling
Xin et al.^145^	Rat BMSC-derived, wt/MiR-133b^−^/MiR-133b^+^	120 min MCAO, male Wistar rats	IA	3 × 10^11^ particles	24 h	Increased functional recovery (mNSS, foot fault), increased neurite remodelling
Xin et al.^146^	Rat BMSC-derived, overexpressing MiR-17-92 cluster	120 min MCAO, male Wistar rats	IV	100 µg	24 h	Increased functional recovery (mNSS, foot fault), neurite remodelling, neurogenesis and oligodendrogenesis

AMSCs: adipose-derived mesenchymal stem cells BMSCs: bone marrow-derived mesenchymal stem cells; EVs: extracellular vesicles; MCAO: middle cerebral artery occlusion; mNSS: modified neurological severity score; MWM: Morris water maze; MVs: microvesicles; SCI: subcortical infarct; UMSCs: umbilical cord-derived mesenchymal stem cells.

## Conclusions and future directions

**Figure 2.**
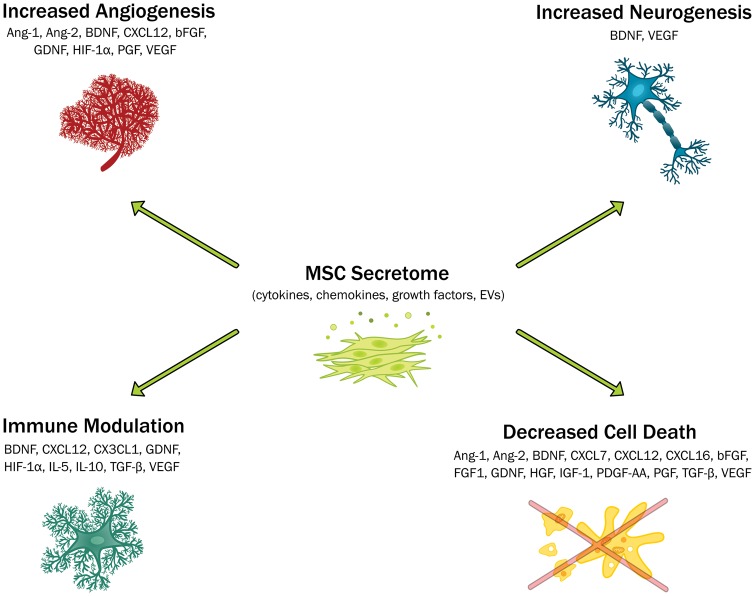
The role of the mesenchymal stem cell secretome in promoting repair and recovery after ischaemic stroke. The main mechanisms of action are highlighted along with the proposed mediators. Ang: angiopoietin; BDNF: brain-derived neurotrophic factor; CXCL: chemokine C-X-C motif ligand; CX3CR1: CX3C chemokine receptor 1; bFGF: basic fibroblast growth factor; GDNF: glial cell line-derived neurotrophic factor; HGF: hepatocyte growth factor; HIF-1α: Hypoxia-inducible factor 1-alpha; IGF-1: insulin-like growth factor 1; IL: interleukin; PDGF-AA: platelet-derived growth factor AA; PGF: placental growth factor; TGF-β: transforming growth factor beta: VEGF: vascular endothelial growth factor.

There is a growing body of evidence demonstrating the role of the MSC secretome in promoting recovery in rodent models of cerebral ischaemia. This has been proposed to occur through a number of mechanisms including decreased neuroinflammation, neuroprotection, increased angiogenesis and neurogenesis (Figure 2). However, there is currently no consensus in the literature on what mediators in the MSC secretome are important in promoting repair and functional recovery after stroke. While a strong case can be made for BDNF in particular with multiple citations supporting its role, neutralising BDNF did not completely abolish post-stroke recovery. It is therefore likely that a combination of mediators is important in promoting recovery and repair after stroke. In support of this, meta-analysis has demonstrated that G-CSF does not improve outcomes in stroke patients.^148^ A number of in vitro strategies have been used to drive the secretome towards a more desirable anti-inflammatory and pro-trophic phenotype including priming with pro-inflammatory cytokines, hypoxic preconditioning, biomaterials and 3D culture (Figure 1). However, the efficacy of these approaches has not been extensively assessed in preclinical models.

There are several challenges to be overcome in translating the MSC secretome into a safe and effective therapy for ischaemic stroke such as the optimal timing of administration. The majority of preclinical studies elected to administer MSCs, CM and exosomes during the acute phase of stroke (≤ 48 h) where secondary damage is mediated by reactive oxygen species, migration of immune cells to the ischaemic brain and production of pro-inflammatory cytokines such as IL-1.^149^ As a number of studies have demonstrated immunomodulatory and neuroprotective effects of the MSC secretome, such a time point may hold therapeutic potential. In contrast, one study reported that administration of MSCs to rats at 1 month post-stroke also led to functional recovery. This was associated with decreased glial scarring and increased proliferating cells in the subventricular zone, suggesting MSC treatment may have promoted neurogenesis.^150^ As MSCs secrete multiple growth factors which can activate endogenous repair mechanisms, administration at delayed time points should be investigated further.^151^ Determining the optimal timing of administration may prove to be a difficult balancing act and repeated dosing should be considered. For example, VEGF induces vascular permeability so if administered at acute time points may increase BBB breakdown leading to increased cerebral oedema and exacerbate injury. Another challenge will be determining the best therapy. While MSCs are generally immune evasive and have been shown to be well tolerated in clinical trials in stroke, the increasing number of preclinical studies demonstrating the efficacy of MSC-derived CM and EVs could mitigate the need to administer cells. This may prove more translatable as these cell-free alternatives can be cryopreserved without any concerns over cell viability so could be stored for long periods of time and shipped worldwide. Another challenge will be determining the route of administration. Preclinical studies and clinical trials have employed both systemic routes such as IV and IV and direct routes such as IC. As improvements in recovery can occur independently of MSC engraftment or even migration to the ischaemic brain, perhaps systemic routes which are simpler, less invasive and less likely to cause adverse events should be adopted.

Looking forward, the biggest challenge to preclinical scientists is that there is currently no clear consensus on the optimum culture conditions and preconditioning strategy to maximise the regenerative potential of the MSC secretome. Future work should focus on assessing the efficacy of more approaches to modifying secretome in rodent models of cerebral ischaemia and increasing our understanding of the mediators involved in promoting repair. There is growing interest in cell-free approaches such as exosomes or CM and these should also be more fully investigated. In summary, while there are a number of hurdles to overcome on road to translation, the MSC secretome holds much potential as a regenerative therapy for ischaemic stroke.
